# Analytical and Clinical Validation of a Plasma Fibroblast Growth Factor 21 ELISA Kit Using an Automated Platform in Steatotic Liver Disease

**DOI:** 10.3390/biom15060877

**Published:** 2025-06-16

**Authors:** Makito Tanaka, Shingo Tanaka, Ryo Kobayashi, Ryosei Murai, Satoshi Takahashi

**Affiliations:** 1 Division of Laboratory Medicine, Sapporo Medical University Hospital, Sapporo 060-8543, Japan; mkt-tnk@sapmed.ac.jp (M.T.); r.kobayashi@sapmed.ac.jp (R.K.); r.murai@sapmed.ac.jp (R.M.); stakahas@sapmed.ac.jp (S.T.); 2 Department of Infection Control and Laboratory Medicine, Sapporo Medical University School of Medicine, Sapporo 060-8543, Japan

**Keywords:** attenuation imaging, enzyme-linked immunosorbent assay, fibroblast growth factor 21, noninvasive diagnostic biomarker, sample stability, steatotic liver disease

## Abstract

Steatotic liver disease is a global health challenge that requires reliable and noninvasive diagnostic biomarkers. This research aimed to validate the analytical and clinical performance of a fibroblast growth factor 21 (FGF21) enzyme-linked immunosorbent assay (ELISA) kit using an automated immunoassay analyzer. Plasma FGF21 levels were measured using a commercial ELISA kit on an automated immunoassay analyzer. Validation included intra- and inter-assay precision, dilution linearity, spike recovery, lower limit of quantification (LLOQ), interference testing, and sample stability analysis. Clinical evaluation involved 97 patients who underwent abdominal ultrasound-based attenuation imaging for the diagnosis of hepatic steatosis. The assay demonstrated high analytical precision, with intra- and inter-assay coefficients of variation <15% and an LLOQ of 3.260 pg/mL. Dilution linearity, spike recovery, and interference tests confirmed the reliability of the assay, whereas stability tests highlighted the minimal effect of freeze-thaw cycles and storage conditions. Clinically, FGF21 levels correlated with attenuation coefficient (*r* = 0.44). Diagnostic performance indicated 84% sensitivity and 81% specificity at defined FGF21 thresholds for the diagnosis of hepatic steatosis. This research confirmed the reliable analytical and clinical performance of the FGF21 ELISA kit, reinforcing its potential as a diagnostic biomarker of hepatic steatosis.

## 1. Introduction

Steatotic liver disease (SLD) is a prevalent condition, impacting nearly 30% of the worldwide population, with primary contributing factors including obesity, type 2 diabetes, and alcohol consumption [1]. Recently, a novel classification of SLD, termed metabolic dysfunction-associated steatotic liver disease (MASLD), has been proposed [2]. Similar with the previous definition of non-alcoholic fatty liver disease (NAFLD), the diagnosis of hepatic steatosis remains essential and includes subcategories addressing all potential causes of liver steatosis [2]. Although abdominal ultrasonography (US) is considered the first-choice modality for diagnosing steatosis, recent advancements have introduced quantitative attenuation-based techniques for assessing hepatic fat content, which are now increasingly used in clinical practice [3,4].

MASLD has been identified as a risk factor not only for liver-related complications, such as cirrhosis and hepatocellular carcinoma, but also for systemic conditions, including cardiovascular disease and malignancies [5]. Therefore, early detection of MASLD is of critical importance. The global prevalence of MASLD is increasing, particularly in developing countries [6]. In these regions, limited medical resources make it impractical to perform US on many individuals [7]. Consequently, there is an urgent need for alternative diagnostic approaches to identify hepatic steatosis that do not rely on imaging modalities. The development of reliable and accessible biomarkers is highly anticipated to address this challenge.

Fibroblast growth factor 21 (FGF21) is a non-canonical member of the fibroblast growth factor family that functions as an endocrine hormone. It is predominantly produced in the liver under physiological conditions and plays a critical role in the regulation of metabolic homeostasis [8]. FGF21 is known for its ability to modulate energy expenditure, improve insulin sensitivity and regulate glucose and lipid metabolism [8]. Furthermore, an FGF21 analog is currently used in the treatment of MASLD [9]. Consequently, although numerous studies have reported the utility of circulating FGF21 levels as a biomarker, commonly measured using an enzyme-linked immunosorbent assay (ELISA) [10], evaluations of the assay performance and stability of FGF21 measurements remain limited.

An association between SLD and FGF21 has been demonstrated in some clinical studies [11,12,13] and proteomic analyses [14]. However, the relationship between FGF21 levels and abdominal ultrasound attenuation techniques, which are novel diagnostic methods for steatotic liver, has not been sufficiently investigated. In this research, we evaluated the performance of a commercial ELISA kit for FGF21 measurement and investigated its potential as a blood-based biomarker for the diagnosis of hepatic steatosis. Based on the results of this study, we believe that the reliability of FGF21 as a biomarker is strengthened. Additionally, this research provides new insights into the utility of FGF21 in the diagnosis of SLD.

## 2. Materials and Methods

### 2.1. Assay Validation

#### 2.1.1. Protocol for Automated FGF21 ELISA Assay

FGF21 concentrations in plasma were measured using an established ELISA kit (Quantikine ELISA Human FGF-21 Immunoassay [DF2100; R&D Systems Inc., Minneapolis, MN, USA]) according to the manufacturer’s instructions (working range: 31.3–2000 pg/mL). An Evolis automated immunoassay analyzer (Bio-Rad, Hercules, CA, USA) was used as the measurement instrument. Once the samples and reagents were loaded in the Evolis system, all subsequent steps were performed automatically according to a programmed protocol. Plasma for measurement is a residual specimen used in medical practice and stored in cryotubes at −80 °C until measurement. Lithium heparin-containing blood collection tubes were used for plasma preparation. The sample analyzer was blinded to the clinical information.

The following were evaluated in accordance with previously published protocols [15].

#### 2.1.2. Intra-Assay Precision

To assess intra-assay precision, twenty samples with three concentration levels (low, medium, and high) were prepared and analyzed in a single experimental run. The within-run coefficient of variation (CV) was calculated to determine the precision.

#### 2.1.3. Inter-Assay Precision

For inter-assay precision, twenty-five samples with three concentration levels of FGF21 were analyzed on five separate occasions (5 × 5). The CV between different runs was calculated to assess reproducibility. Precision criteria were met if the CV was <15% [16].

#### 2.1.4. Lower Limits of Quantification (LLOQ)

To determine the LLOQ, sixteen blank samples were analyzed, and the mean signal plus ten times the standard deviation (SD) was used to interpolate the minimum quantifiable FGF21 concentration [15]. To evaluate precision at the LLOQ, twenty low-concentration samples near the estimated LLOQ were prepared and analyzed within a single experimental run. The CV was calculated to assess the precision.

#### 2.1.5. Dilution Linearity

A high-concentration FGF21 sample was serially diluted (×2, ×4, ×8, ×16, ×32, ×64, ×128, ×256, and ×512) with the sample diluent until it exceeded the assay’s linear range. Each diluted sample was tested in duplicate, with results adjusted according to the dilution factor. Recovery at each dilution was calculated using the formula:

%Recovery = [(Measured concentration × diluted factor)/Theoretical concentration] × 100

#### 2.1.6. Spike Recovery Tests

Three plasma samples with different FGF21 levels were divided into three aliquots each and supplemented with 0, 100, and 200 pg of FGF21 standard in 100 μL of solution (containing patient plasma, sample buffer, and spiked peptide). Each of the nine aliquots (3 × 3) was tested in duplicate within the same run. The recovery percentage was calculated using the formula:

%Recovery = ([Measured concentration _spiked sample_ − Measured concentration _neat sample_]/Theoretical concentration _spiked_) × 100

#### 2.1.7. Interference Assessment

Interference testing was performed using bilirubin (conjugated and unconjugated), hemoglobin, intralipid (Interference Check A Plus, Sysmex, Kobe, Japan), and rheumatoid factor (RF; Interference Check RF Plus, Sysmex, Kobe, Japan), following manufacturer’s instructions. The highest concentrations tested were 20 mg/dL for bilirubin, 510 mg/dL for hemoglobin, 1420 FTU for intralipid, and 50 IU/mL for rheumatoid factor (RF). Each interferent was evaluated independently by mixing human plasma with the respective interfering substance using a six-point dilution approach. The relative bias was calculated by comparing observed and baseline values.

#### 2.1.8. Comparison of FGF21 Concentrations Among Different Blood Collection Tubes

To assess the consistency of blood FGF21 concentrations across different blood collection tubes, we compared samples collected using three tube types: lithium heparin, EDTA, and serum tubes. Blood was drawn simultaneously into all three types of tubes from each of 18 patients. Residual specimens obtained from clinical practice were used. Plasma and serum samples were aliquoted and stored at −80 °C until measurement. All measurements were performed in a single run.

#### 2.1.9. Sample Stability Evaluation

Sample stability was assessed under three conditions:

(1) Freeze-thaw stability (Supplementary Figure S1A): Plasma samples were subjected to multiple freeze-thaw cycles (1, 2, 3, 5, and 7 cycles) while being stored at −80 °C between cycles. A reference aliquot remained at −80 °C without undergoing thawing. Each cycle consisted of thawing at room temperature for 2 h, followed by at least 12 h of storage at −80 °C before the next cycle.
(2) Room temperature and refrigerated stability (Supplementary Figure S1B): Plasma samples were kept at room temperature or 4 °C for varying time intervals (1 h, 2 h, 4 h, 24 h, 3 days, and 7 days).
(3) Long-term storage stability (Supplementary Figure S1C): One aliquot was stored at −80 °C as a reference, while others were maintained at −20 °C for one month.
  After the designated storage and freeze-thaw processes, all the samples were stored at −80 °C for further analysis.

### 2.2. Clinical Validation

#### 2.2.1. Study Population

This retrospective cohort study analyzed the data of outpatients who visited our hospital between April 2023 and January 2024. Eligibility criteria were as follows: age 20–69 years; body mass index (BMI) < 30 kg/m^2^; FIB-4 index < 2.67 (to eliminate individuals at high risk of advanced liver fibrosis); alcohol consumption meeting the MASLD criteria [2] (weekly alcohol intake of <140 g for females and <210 g for males [average daily intake of <20 g for females and <30 g for males]); abdominal US and blood sampling performed on the same day; fasting for at least 6 h before the examination; and no liver disease other than SLD diagnosed or treated, such as viral hepatitis or drug-induced liver injury.

#### 2.2.2. Abdominal US Examination

All abdominal US procedures were performed using an Aplio i700 scanner (Canon Medical Systems, Otawara, Japan) with a 1–8 MHz convex transducer (PVI-475BX). Following a routine B-mode ultrasound examination, attenuation imaging (ATI) and two-dimensional shear-wave elastography (SWE) were performed through the rib space to the right lobe of the liver. For the ATI, a fan-shaped sample box was positioned over the hepatic parenchyma, and a 2 × 4 cm measurement region of interest (m-ROI) was defined. The m-ROI was centered on the image, ensuring the avoidance of vessels and shadowing artifacts. Its upper edge was positioned just below the multi-reflection layer in accordance with the vendor’s instructions (Supplementary Figure S2). The SWE measurements were performed according to previously established protocols [17].

#### 2.2.3. Clinical and Laboratory Data

Baseline parameters of each patient, such as sex, age, height, weight, and daily alcohol intake, were recorded on the day of examination. In all cases, the blood concentrations of aspartate aminotransferase (AST), alanine aminotransferase (ALT), γ-glutamyl transpeptidase (γ-GT), total bilirubin, albumin, and the platelet count were measured using standard biochemical methods at our hospital laboratory. The fibrosis-4 (FIB-4) index was determined using the formula [18]:

FIB-4 index = (AST × Age)/(Platelet count × √ALT).

### 2.3. Statistical Analysis

All statistical analyses were conducted using Microsoft Excel for Mac (version 16.93, Microsoft, Redmond, WA, USA) and JMP Pro (version 17.0.0, SAS, Cary, NC, USA). To compare groups, the Mann-Whitney U test was employed for unpaired quantitative variables, and the Wilcoxon signed-rank test was used to compare paired samples. Associations between continuous variables were assessed using Spearman’s rank correlation coefficient. Receiver operating characteristic (ROC) curve analysis was performed to evaluate the diagnostic accuracy of plasma FGF21 concentrations for hepatic steatosis. The area under the curve (AUC) was calculated. Based on the ROC analysis, two cut-off values were established: one with high sensitivity for ruling out steatosis, and another with high specificity for confirming the diagnosis. Sensitivity, specificity, positive likelihood ratio, and negative likelihood ratio were calculated to evaluate the diagnostic performance for steatotic liver [19]. All statistical tests were two-tailed, and a *p*-value < 0.05 was considered statistically significant.

To evaluate the certainty and stability of the analytes, the total change limit (TCL) was determined using the equation:

TCL = ([2.77 × CV_a_]^2^ + [0.5 × CV_b_]^2^)^0.5^

where CV_a_ represents analytical imprecision and CV_b_ denotes within-run variability [20]. If the mean percentage difference for an analyte exceeded the calculated TCL, it was regarded as statistically significant, indicating that the variability did not meet the predefined criteria.

## 3. Results

### 3.1. Assay Validation

#### 3.1.1. Intra- and Inter-Assay Precision

The summary of intra- and inter-assay precision results is shown in Table 1. For intra-assay precision, the CV values for the low, medium, and high concentration samples were 5.4%, 3.2%, and 5.2%, respectively. These results indicate stable performance of the assay within a single run, as reflected by relatively low CV values.

Regarding inter-assay precision, the CV values observed for the low, medium, and high concentration samples were 8.6%, 5.1%, and 8.0%, respectively. Observed CV values indicated that the assay maintained acceptable precision among different assay runs.

#### 3.1.2. Lower Limits of Quantification (LLOQ)

The LLOQ for this assay was 3.260 pg/mL, calculated as the interpolated FGF21 concentration, based on the mean signal plus 10 SD of the blank samples [15]. To assess precision at the LLOQ, near the estimated LLOQ were analyzed in a single experimental run. The CV was 3.7%, with a mean measured concentration of 2.870 pg/mL.

#### 3.1.3. Dilution Linearity

Dilution linearity of the assay is shown in Table 2. The observed concentrations were in close agreement with the expected values across dilution factors ranging from ×2 to ×128, with recovery rates between 97.3% and 110.8%. The CV remained below 6% within this range, and the deviation from the mean values did not exceed the TCL (14%). These results indicate that the assay maintained acceptable accuracy and precision across the working range recommended by the manufacturer. However, beyond a 128-fold dilution, the assay appeared to lose linearity, likely due to excessive sample dilution rather than insufficient assay sensitivity.

#### 3.1.4. Spike Recovery Tests

Spike recovery test results for the three samples are presented in Table 3. Recovery rates across all the samples and spike levels were consistently within an acceptable range of 95.9–99.7%, demonstrating a high degree of accuracy in quantifying spiked analyte concentrations for the assay. The low CV values further indicate a high level of precision in the measurements.

#### 3.1.5. Interferences

The interference of bilirubin (conjugated and free), hemoglobin, chyle, and rheumatoid factor are shown in Supplementary Figure S3. Across all panels, none of the results exceeded the TCL, indicating that all the measurements remained within an acceptable range. These findings suggest that the assay is resistant to potential interfering substances, maintaining consistent performance across varying concentrations.

#### 3.1.6. Correlation and Comparison of FGF21 Levels Between Tube Types

**Figure 1:**
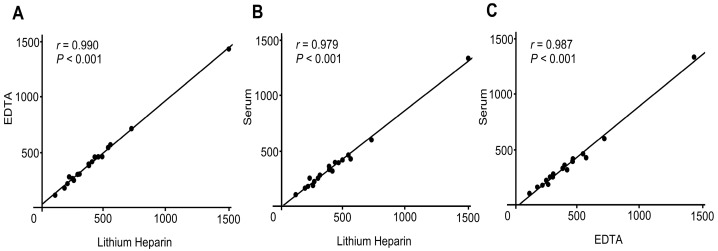
Comparison of FGF21 concentrations measured using lithium heparin, EDTA, and serum tubes: (**A**) Lithium heparin vs. EDTA, y = 0.941x + 27.2; (**B**) Lithium heparin vs. serum, y = 0.875x − 8.95; (**C**) EDTA vs. serum, y = 0.927x − 33.2. Spearman’s rank correlation coefficients (*r*) demonstrated strong positive correlations among all tube types. Paired samples were collected simultaneously from 18 patients to allow for direct comparison. Abbreviations: EDTA = ethylenediaminetetraacetic acid.

Spearman’s rank correlation analysis showed strong correlations of FGF21 concentrations between lithium heparin and EDTA (*r* = 0.990; Figure 1A), lithium heparin and serum (*r* = 0.979; Figure 1B), and serum and EDTA (*r* = 0.987; Figure 1C), with all correlations being statistically significant (*p* < 0.001).

Wilcoxon signed-rank test revealed no significant difference between lithium heparin and EDTA tubes (*p* = 0.341). In contrast, serum samples showed significantly lower FGF21 levels compared to both lithium heparin and EDTA samples (*p* < 0.001 for both comparisons), suggesting a matrix effect associated with serum.

#### 3.1.7. Sample Stability

**Figure 2:**
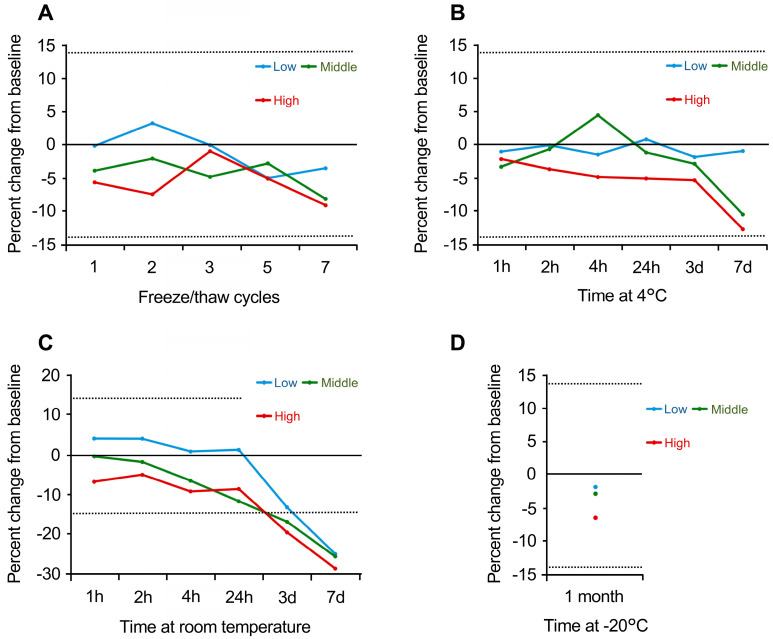
Plasma stability under different storage conditions: (**A**) Freeze/thaw stability: Percentage change in FGF21 levels after 1–7 cycles. (**B**) Refrigeration at 4 °C: Stability assessment over 1 h to 7 days. (**C**) Room temperature stability: stability assessment over 1 h to 7 days (**D**) Long-term freezer stability: samples stored at −20 °C for 1 month. Results indicate minimal degradation, with significant declines observed only after extended room temperature exposure.

Figure 2 illustrates the effect of various environmental conditions such as freeze-thaw cycles, temperature, and duration, on sample stability. In Figure 2A, the percentage change from the initial value after multiple freeze-thaw cycles is presented. The high and medium groups showed a reduction in the initial value of approximately 10% after the seventh cycle, whereas the low group experienced a more gradual decline.

Figure 2B shows the effects of refrigeration at 4 °C. After 24 h, all the groups exhibited a slight decrease in stability. By day seven, the high and medium groups had decreased to nearly −14%, while the low group showed only minimal changes. Figure 2C demonstrates the effects of room temperature on stability, where a sharp drop occurred after 3 days, with values falling by approximately 30% in all the groups by day seven. Figure 2D examines storage at −20 °C, showing that freezing at this temperature maintained relatively stable retention across all concentration levels.

### 3.2. Clinical Validation

**Figure 3:**
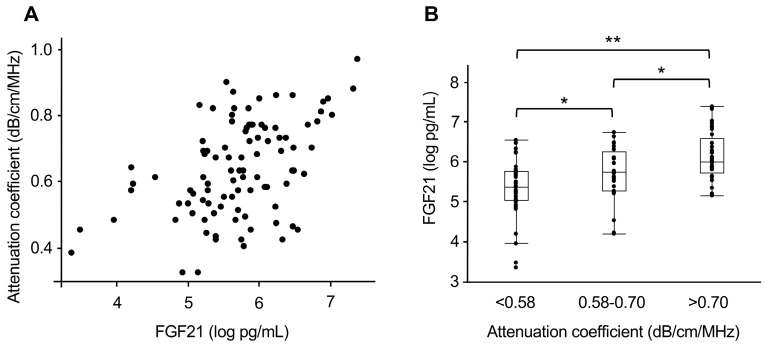
Correlation of FGF21 with steatosis severity: (**A**) Scatter plot of FGF21 levels and attenuation coefficient values obtained via ultrasound. Positive correlation observed (*r* = 0.437, *p* < 0.001). (**B**) FGF21 levels stratified by attenuation coefficient categories: normal (<0.58 dB/cm/MHz; n = 36), suspected steatosis (0.58–0.70 dB/cm/MHz; n = 28), and confirmed steatosis (>0.70 dB/cm/MHz; n = 33). Significant differences in FGF21 levels highlight its potential as a biomarker for steatosis severity. * *p* < 0.05, ** *p* < 0.01.

Baseline clinical characteristics of the 97 patients included in this study are summarized in Table 4. Median age of the patients was 56 years; 48% were males, and median BMI recorded at 22.5 kg/m^2^. Key liver function markers included a median ALT level of 21 U/L and γGT was 21 U/L. Table 5 and Figure 3A show correlations between plasma FGF21 levels and various clinical parameters. A significant positive correlation was identified between plasma FGF21 levels, BMI (*r* = 0.334, *p* < 0.001), and attenuation coefficient (*r* = 0.437, *p* < 0.001).

Figure 3B shows FGF21 concentrations according to three categories of steatotic liver diagnosis: attenuation coefficient < 0.58 dB/cm/MHz (normal), 0.58–0.70 dB/cm/MHz (suspected steatotic liver), and > 0.70 dB/cm/MHz (confirmed steatotic liver). These classifications were based on previously established cut-off values [3], which were originally developed to differentiate between the presence and absence of hepatic steatosis (i.e., grade 0 vs. grade ≥1). Significant differences were observed in each category, indicating a relationship between the degree of hepatic fat deposition and plasma FGF21 levels.

For the diagnosis of steatotic liver (with an attenuation coefficient cut-off of 0.66 [21]), ROC curve analysis demonstrated an AUC of 0.727 for plasma FGF21 in detecting hepatic steatosis. Based on this, two clinically meaningful thresholds were selected. FGF21 level < 251 pg/mL was found to be a reliable threshold to rule out hepatic steatosis (sensitivity = 84%; negative likelihood ratio = 0.3). Conversely, FGF21 level ≥ 440 pg/mL was useful for confirming the condition, with a specificity of 81% and a positive likelihood ratio of 2.2.

## 4. Discussion

This study validates the analytical and clinical performance of a commercial FGF21 ELISA kit for assessing hepatic steatosis. Various analytical parameters were evaluated following previously established protocols [15]. The findings confirmed that the assay provides high sensitivity for detecting FGF21 in human plasma, with an LLOQ of 3.260 pg/mL. The intra- and inter-assay precision, expressed as %CV, remained below 15% (Table 1), meeting the recommended criteria for biomarker validation [16]. Furthermore, assessments of dilution linearity (Table 2), spike recovery (Table 3), and interference (Supplementary Figure S3) demonstrated satisfactory outcomes. Additionally, comparison of FGF21 levels between tube types (Figure 1) and detailed stability analyses were conducted (Figure 2), which yielded numerous new findings.

Blood FGF21 level is widely used as a biomarker in clinical studies, particularly in metabolic syndrome. Although most studies use ELISA for measurement, detailed descriptions of the measurement procedures and performance evaluations are rare. However, ELISA is often performed manually in many facilities, which can lead to significant variability in measurements. In contrast, this study used a fully automated system that offers distinct advantages. According to Rohner et al. [22], automated ELISA systems provide improved precision, reduced operator dependence, and faster sample throughput than the manual methods. The reliable analytical performance demonstrated in this study was likely due to the use of this automated system. When measuring the FGF21 levels in large sample sets, the adoption of a fully automated system is strongly recommended to ensure accuracy and efficiency.

In our study, plasma FGF21 concentrations measured in heparinized and EDTA-treated samples were comparable. In contrast, serum samples showed significantly lower FGF21 levels. This discrepancy is likely attributable to proteolytic degradation of FGF21 during the clotting process, as serum preparation involves incubation at room temperature, allowing activation of proteases such as fibroblast activation protein, which cleaves FGF21 at its C-terminus and diminishes its detectability [23]. Our findings suggest that plasma samples are more suitable for reliable quantification of circulating FGF21.

In recent years, biobank materials have been widely used in medical research, with sample quality being a critical factor. In the case of biobanks, limitations in storage space and sample volume may make aliquoting difficult, potentially necessitating multiple freeze-thaw cycles for the same sample. It is assumed that multiple freeze-thaw cycles may affect the measurement of biomarkers in archival samples [24,25]. The manufacturer’s manual for the ELISA kit used in this study included a recommendation to “avoid repeated freeze-thaw cycles”. However, to the best of our knowledge, no studies have examined the effect of freeze-thaw cycles. This study demonstrated that FGF21 is highly stable against freeze-thaw cycles, with changes of <10% observed even after seven cycles (Figure 2A). In clinical trials using stored samples, freeze-thaw processes are often unavoidable. Therefore, the finding that FGF21 is relatively unaffected by freeze-thaw cycles represents a significant and valuable insight.

Currently, histological findings and MRI derived proton density fat fraction are considered the gold standards for diagnosing steatotic liver. However, recent studies suggest that the US attenuation method demonstrates a diagnostic accuracy comparable to that of these methods, particularly when excluding obese individuals [26], as was done in this study. In this study, we examined the relationship between plasma FGF21 levels and attenuation coefficient through ATI, revealing a relatively strong correlation (Figure 3A). Although previous reports have demonstrated a close association between FGF21 and liver fat content [11,12,13], these studies primarily relied on semi-quantitative methods, such as histological findings or B-mode US. In contrast, this study used the attenuation coefficient, a highly quantitative method for evaluating steatotic liver, allowing for a more detailed investigation of its relationship with FGF21 levels.

A critical limitation of using FGF21 as a biomarker for SLD is its susceptibility to various factors other than metabolic conditions [10]. Notably, FGF21 level is known to increase with age [27,28,29], liver fibrosis [30], and habitual alcohol consumption [31,32]. In this study, we excluded individuals aged ≥70 years, those with advanced liver fibrosis (FIB-4 index ≥ 2.67), and habitual drinkers, yielding favorable results. In our cohort, no significant correlation was observed between age and plasma FGF21 levels (*r* = 0.061, *p* = 0.551; Table 5). Although previous studies have reported that FGF21 levels increase with age [27,28,29], this effect may not have been prominent in our study due to the exclusion of elderly individuals aged ≥70 years. Given that such cases are likely to be excluded in routine health checkups, our findings suggest the potential utility of FGF21 for screening SLD in health screening settings.

Numerous studies have been conducted to diagnose steatotic liver using blood biomarkers and scoring systems. Among these, the Fatty liver index (FLI) [33] has been the most extensively studied. The FLI is calculated based on BMI, waist circumference, γ-GT, and triglyceride levels, and has been widely investigated, particularly in health screening settings. In terms of diagnostic performance, an FLI of <30 effectively rules out steatotic liver, achieving a sensitivity of 87% and a negative likelihood ratio of 0.2. Conversely, an FLI of ≥60 is commonly used to confirm steatotic liver, with a specificity of 86% and a positive likelihood ratio of 4.3 [33]. In our study, FGF21 alone demonstrated diagnostic performance comparable to that of FLI in terms of sensitivity, specificity, and negative likelihood ratio. However, the positive likelihood ratio of FGF21 expression was lower than that of FLI. Nevertheless, our findings strongly indicate that FGF21 has significant potential as a diagnostic marker for SLD. Furthermore, combining FGF21 with other biomarkers may result in a diagnostic performance that surpass that of FLI.

One limitation of this study is that it was conducted at a single center, which may restrict the generalizability of the clinical findings. Larger multicenter prospective studies are warranted to validate these results. Furthermore, all the participants in this study were Japanese. Given that ethnicity can influence various diseases, additional studies involving diverse ethnic populations is necessary to ensure the broader applicability of these findings. Moreover, data on insulin resistance and lipid profiles, which have been shown in previous studies to be associated with circulating FGF21 levels, were not available in our dataset. Due to the retrospective nature of the study, these metabolic parameters could not be assessed, limiting our ability to interpret the metabolic context of FGF21 in greater detail.

In addition, cross-reactivity and non-specific binding were not experimentally evaluated in our laboratory, primarily due to cost constraints. However, according to the manufacturer’s documentation, the ELISA kit used in this study has been tested for cross-reactivity against multiple related molecules, with no significant cross-reactivity observed. Independent evaluation of these aspects may further strengthen the analytical validity of the assay.

## 5. Conclusions

This study demonstrates the reliable analytical and diagnostic performance of a commercial FGF21 ELISA kit when measured using an automated immunoassay analyzer. Furthermore, the stability assessment demonstrated that plasma FGF21 levels were minimally affected by repeated freeze-thaw cycles and various storage conditions. These findings enhance the credibility of previous clinical studies utilizing plasma FGF21 measurements. Although the diagnosis of steatotic liver based solely on FGF21 may be challenging, the combination of FGF21 with other biomarkers shows promise for enabling a non-imaging-based approach for the diagnosis of steatotic liver.

## Figures and Tables

**Table 1:** Result of intra and inter-assay precision.

	Intra-Assay Precision			Inter-Assay Precision		
Sample	Low	Medium	High	Low	Medium	High
Number	20	20	20	25	25	25
Mean (pg/mL)	159	579	1177	207	607	1020
Standard deviation	8.5	18.6	61.7	17.8	31.1	81.9
CV (%)	5.4	3.2	5.2	8.6	5.1	8.0

Abbreviations: CV = coefficient of variation.

**Table 2:** Dilution linearity of FGF21 assay.

Fold Dilution	Expected (pg/mL)	Observed (pg/mL)	Recovery (%)	CV (%)
×2	1500	1508.6	100.6	0.9
×4	750	793.75	105.8	0.3
×8	375	388.05	103.5	2.2
×16	187.5	182.4	97.3	1.5
×32	93.8	94.7	101.0	1.2
×64	46.9	51.95	110.8	0.1
×128	23.4	23.65	101.1	5.7
×256	11.7	5.9	50.4	28.8
×512	5.9	2.7	45.8	55.6

Abbreviations: CV, coefficient of variation.

**Table 3:** Recovery rate for each sample in the spike recovery tests.

	Spike	Measured Conc. (pg/mL)	CV (%)	Theoretical Conc. (pg/mL)	Recovery Rate (%)
Sample 1	0 pg	164	2.3	–	–
	100 pg	261	1.4	264	98.9
	200 pg	360	0.6	364	98.9
Sample 2	0 pg	220	1.4	–	–
	100 pg	319	3.2	320	99.7
	200 pg	413	1.2	420	98.3
Sample 3	0 pg	392	2.7	–	–
	100 pg	483	1.2	492	98.2
	200 pg	568	1.0	592	95.9

Abbreviations: Conc. = concentration; CV = coefficient of variation.

**Table 4:** Baseline clinical characteristics of patients (n = 97).

Age, Years	56	(27–69)
Male	47	(48)
BMI, kg/m^2^	22.5	(15.4–29.3)
AST, U/L	20	(13–67)
ALT, U/L	21	(7–134)
γ-GT, U/L	21	(6–414)
Total bilirubin, mg/dL	0.8	(0.3–2.8)
Albumin, g/dL	4.3	(3.6–5.1)
Platelet count, ×10^4^ μL	23.3	(13.9–46.6)
FIB-4 index	1.13	(0.40–2.28)
Shear-wave speed, m/sec	1.24	(0.94–1.58)
Attenuation coefficient, dB/cm/MHz	0.63	(0.32–0.97)

Data are presented as n (%) or median (range). Abbreviations: BMI = body mass index; AST = aspartate transaminase; ALT = alanine transaminase; γ-GT = γ-glutamyl transpeptidase; FIB-4 = fibrosis-4. FIB-4 index = (AST × Age)/(Platelet count × √ALT).

**Table 5:** Correlations between clinical findings and FGF21 levels (n = 97).

Clinical Parameters	Statistics	
	*r*	*p* Value
Age, years	0.061	0.551
BMI, kg/m^2^	0.334	<0.001
AST, U/L	0.053	0.610
ALT, U/L	0.089	0.385
γ-GT, U/L	0.192	0.059
Total bilirubin, mg/dL	−0.159	0.119
Albumin, g/dL	−0.039	0.702
Platelet count, ×10^4^/μL	0.200	0.049
FIB-4 index	−0.111	0.279
Shear-wave speed, m/s	0.036	0.724
Attenuation coefficient, dB/cm/MHz	0.437	<0.001

Abbreviations: *r* = correlation coefficient; BMI = body mass index; AST = aspartate transaminase; ALT = alanine transaminase; γ-GT = γ-glutamyl transpeptidase; FIB-4 = fibrosis-4. index = (AST × Age)/(Platelet count × √ALT).

## Data Availability

The original contributions presented in this study are included in the article/supplementary material. Further inquiries can be directed to the corresponding authors.

## Supplementary Materials

The following supporting information can be downloaded at: https://www.mdpi.com/article/10.3390/biom15060877/s1, Figure S1: Sample stability under various conditions; Figure S2: Ultrasound imaging for steatosis evaluation; Figure S3: Interference of substances on FGF21 measurements.

## Abbreviations

The following abbreviations are used in this manuscript:

ALT	alanine aminotransferase
AUC	area under the curve
AST	aspartate aminotransferase
ATI	attenuation imaging
BMI	body mass index
CV	coefficient of variation
EDTA	ethylenediaminetetraacetic acid
ELISA	enzyme-linked immunosorbent assay
FGF21	fibroblast growth factor 21
FIB-4	fibrosis-4
LLOQ	lower limit of quantification
MASLD	metabolic dysfunction-associated steatotic liver disease
NAFLD	non-alcoholic fatty liver disease
RF	rheumatoid factor
ROC	receiver operating characteristic
SD	standard deviation
SLD	steatotic liver disease
SWE	shear-wave elastography
TCL	total change limit
US	ultrasonography
