# Supplementary material for: Analytical and Clinical Validation of a Plasma Fibroblast Growth Factor 21 ELISA Kit Using an Automated Platform in Steatotic Liver Disease
Source: Biomolecules. 2025 Jun 16;15(6):877. doi: 10.3390/biom15060877 (PMC12190780; doi:10.3390/biom15060877)
Supplement: Supplementary file 1 [file biomolecules-15-00877-s001.zip › biomolecules-3651881-supplementary.pdf]

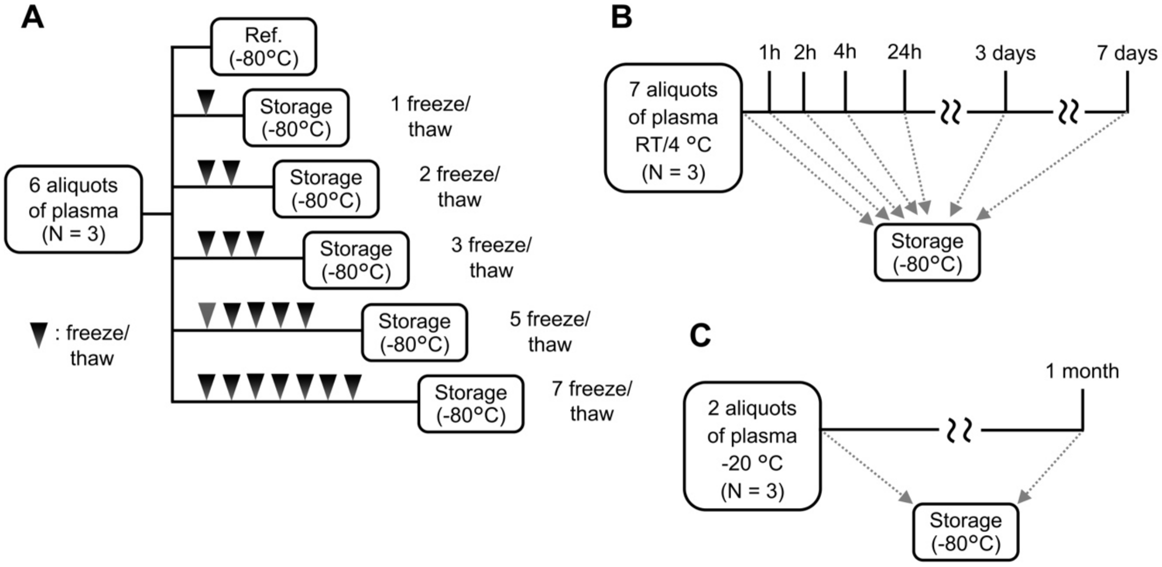

Supplementary Figure S1. Sample stability under various conditions

(**A**) Freeze/thaw stability: plasma samples subjected to 1, 2, 3, 5, and 7 freeze-thaw cycles, with reference stored at −80°C without any freeze-thaw cycle. (**B**) Stability at room temperature (RT) or 4°C: plasma samples stored at RT or 4°C for durations of 1 h to 7 days before freezing at −80°C. (**C**) Stability at −20°C: plasma samples stored at −20°C for 1 month compared to a reference sample stored at −80°C.

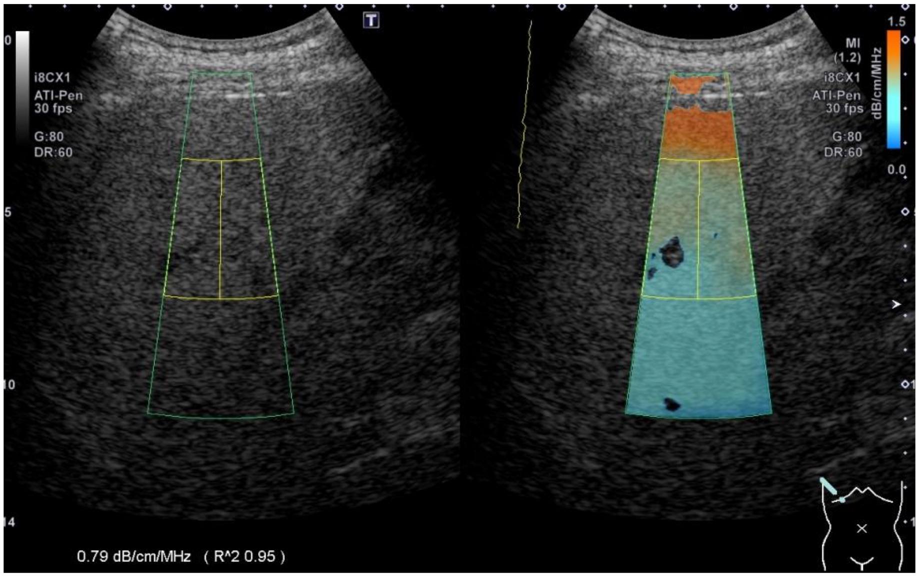

Supplementary Figure S2. Ultrasound imaging for steatosis evaluation

Representative ultrasound images illustrating attenuation imaging (ATI) for the assessment of hepatic steatosis. A fan-shaped sample box was placed over the hepatic parenchyma, carefully avoiding vascular structures and shadow artifacts. A 2 × 4 cm region of interest (yellow line) was defined within the parenchymal area, with its upper edge positioned below the multireflection layer, according to the vendor's instructions. This system provides a quality measure for fat quantification, represented by the linear regression coefficient of determination (R^2) displayed at the bottom left. The R^2 value is color-coded to indicate quality: red (<0.70) denotes poor quality, yellow (0.70–0.80) indicates acceptable quality, and white (≥0.90) represents good quality. In this example, the attenuation coefficient is 0.79 dB/cm/MHz, with an R^2 value of 0.95.

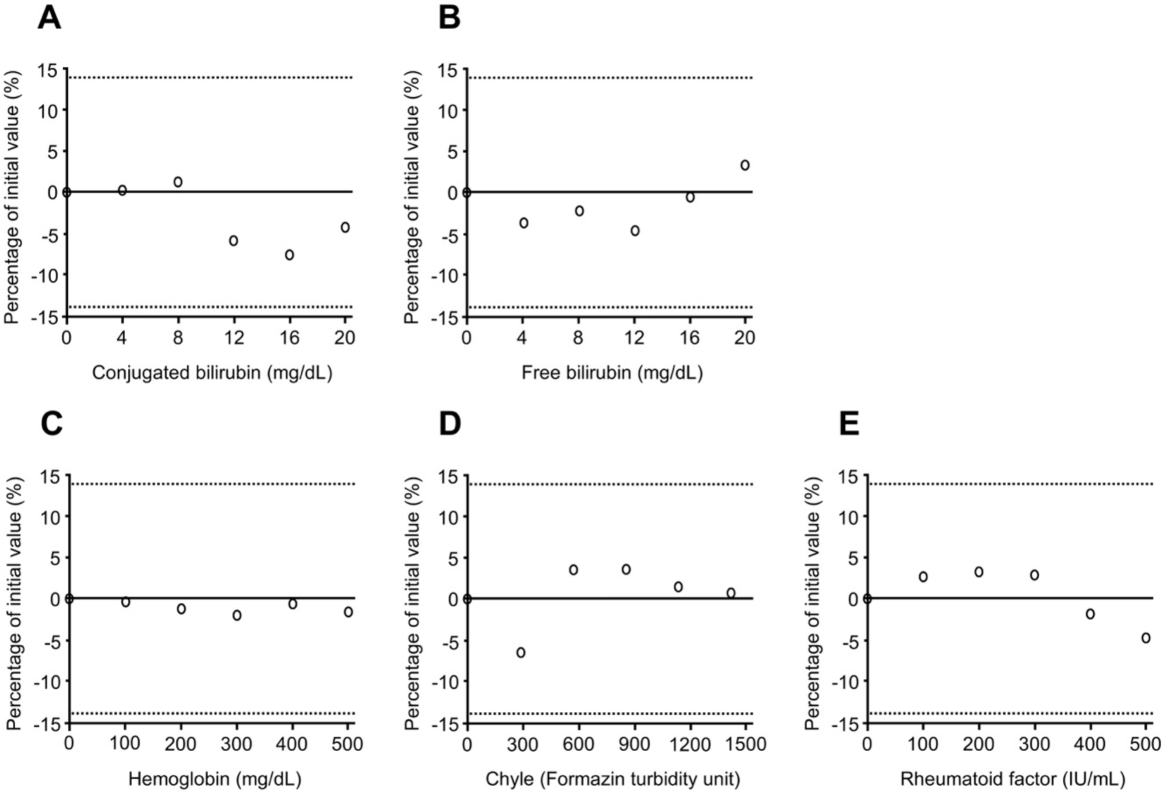

Supplementary Figure S3. Interference of substances on FGF21 measurements

Impact of bilirubin (**A**, Conjugated and **B**, Free forms), hemoglobin (**C**), chyle (**D**), and rheumatoid factor (**E**) on plasma FGF21 measurement. Measurements remained within total change limit (14%) across all tested concentrations, confirming the assay's resistance to interfering substances.
